# An ELISA-based high throughput protein truncation test for inherited breast cancer

**DOI:** 10.1186/bcr2722

**Published:** 2010-10-04

**Authors:** Mark J Lim, Gabriel J Foster, Sadanand Gite, Heather P Ostendorff, Steven Narod, Kenneth J Rothschild

**Affiliations:** 1 AmberGen, Inc., 313 Pleasant Street, Watertown, MA 02472, USA; 2 First Light Biosciences, Inc., 1 Oak Park Drive, Floor 2, Bedford, MA 01730, USA; 3 Centre for Research in Women's Health, Women's College Hospital, University of Toronto, 790 Bay Street, Toronto, ON, M5G 1N8, Canada; 4 Molecular Biophysics Laboratory, Department of Physics and Photonics Center, Boston University, Boston, MA 02215, USA

## Abstract

**Introduction:**

Breast cancer is the most diagnosed and second leading cause of cancer deaths in the U.S. female population. An estimated 5 to 10 percent of all breast cancers are inherited, caused by mutations in the breast cancer susceptibility genes (*BRCA1/2*). As many as 90% of all mutations are nonsense mutations, causing a truncated polypeptide product. A popular and low cost method of mutation detection has been the protein truncation test (PTT), where target regions of *BRCA1/2 *are PCR amplified, transcribed/translated in a cell-free protein synthesis system and analyzed for truncated polypeptides by sodium dodecyl sulfate polyacrylamide gel electrophoresis (SDS-PAGE) and autoradiography. We previously reported a novel High Throughput Solid-Phase PTT (HTS-PTT) based on an enzyme-linked immunosorbent assay (ELISA) format that eliminates the need for radioactivity, SDS-PAGE and subjective interpretation of the results. Here, we report the next generation HTS-PTT using triple-epitope-tagged proteins and demonstrate, for the first time, its efficacy on clinical genomic DNA samples for *BRCA1/2 *analysis.

**Methods:**

Segments of exons 11 of *BRCA1/2 *open reading frames were PCR amplified from either blood derived genomic DNA or cell line mRNA. PCR primers incorporate elements for cell-free transcription/translation and epitope tagging. Cell-free expressed nascent proteins are then antibody-captured onto the wells of a microtiter plate and the relative amount of truncated polypeptide measured using antibodies against the N- and C-terminal epitope tags in an ELISA format.

**Results:**

100% diagnostic sensitivity and 96% specificity for truncating mutations in exons 11 of *BRCA1/2 *were achieved on one hundred blood-derived clinical genomic DNA samples which were previously assayed using the conventional gel based PTT. Feasibility of full gene coverage for *BRCA1/2 *using mRNA source material is also demonstrated.

**Conclusions:**

Overall, the HTS-PTT provides a simple, quantitative, objective, low cost and high throughput method for analysis of truncating mutations as an alternative to gel based PTT for *BRCA *analysis. The technology is readily accessible to virtually any laboratory, with the only major instrumentation required being a PCR thermocycler and a basic micro-well plate reader. When compared to conventional gel based PTT, the HTS-PTT provides excellent concordance.

## Introduction

Breast cancer is the most diagnosed and second leading cause of cancer deaths in the US female population, with approximately 200,000 new cases and approximately 40,000 deaths reported annually [1]. Ovarian cancer is the second ranking gynecological cancer, with approximately 22,000 cases and approximately 15,000 deaths annually [1]. Approximately 3% to 5% of all breast cancers and approximately 10% of ovarian cancers are due to inherited mutations in the breast and ovarian cancer susceptibility 1 or 2 genes (*BRCA1 *or *BRCA2*) [2-4]. Lifetime risks of *BRCA1/2 *mutation carriers have been estimated at approximately 80% for breast cancer and approximately 40% and approximately 20% for ovarian cancer (*BRCA1 *and *BRCA2*, respectively) [5].

Nonsense or frameshift mutations, which result in the truncated gene product (and presumably non-functional or dysfunctional BRCA protein), account for approximately 90% of the clinically important *BRCA1/2 *entries in the Breast Cancer Information Core (BIC) database and approximately 50% fall within the large exon 11 (of *BRCA1 *and *BRCA2*) alone [6]. Mutation-specific techniques are rendered impractical because of the sheer number of mutations (more than 850 clinically important mutations can be found in *BRCA1 *and more than 750 can be found in *BRCA2 *[6]).

In addition to inherited breast and ovarian cancers, a variety of inherited diseases, including familial adenomatous polyposis (*APC*) [7], hereditary non-polyposis colon cancer (*MSH2/MLH1*) [8], polycystic kidney disease (*PKD1*) [9], neurofibromatosis (*NF1 *and *NF2*) [10,11], and Duchenne muscular dystrophy (*DMD*) [12], are caused by chain truncations as the primary mode.

Direct automated intra-exonic DNA sequencing is the 'gold standard' scanning method for detection of *BRCA1/2 *mutations (Myriad Genetics, Salt Lake City, UT, USA). A variety of other approaches, including several electrophoretically based assays that essentially resolve the mutant DNA or protein by mobility differences, are possible. These include the protein truncation test (PTT), conformation-sensitive gel electrophoresis (CSGE), and two-dimensional gene scanning (TDGS) as well as variants of these basic methods. Analogous to the electrophoretic methods, denaturing high-performance liquid chromatography (DHPLC) is a highly sensitive method to detect mutant-WT DNA heteroduplexes. High-throughput or multiplexed mutation-specific methods (that is, requiring *a priori *knowledge of possible mutations) such as DNA microarrays and reverse transcription-polymerase chain reaction (RT-PCR) are also possible. A recent review of *BRCA1/2 *mutation detection methods [2] found that, whereas the specificity was essentially 100% for most methods, the sensitivity varied significantly: the sensitivity of the different electrophoretic mobility-based DNA assays ranged from 50% to 100%, that of the PTT was 75%, and DHPLC was the top performer with a sensitivity of 100% in all cases.

However, methods such as DNA sequencing, DNA microarrays, and DHPLC are expensive to perform or require expensive and specialized instrumentation or both. Thus, despite its imperfect sensitivity, the PTT assay is popular for its simplicity, cost-effectiveness, and general accessibility to virtually any laboratory [5]. Conventional PTT begins with PCR amplification of target gene segments from the patient source material (for example, genomic DNA or mRNA from blood). The PCR primer pair incorporates additional sequences into the amplicons required for subsequent protein production, including an RNA polymerase promoter, a Kozak (ribosome-binding) site, and start and stop codons. The amplified DNA is then added to a cell-free transcription-translation extract along with radioactive amino acids (^35^S-methione or ^14^C-leucine), and the expressed protein is then analyzed by SDS-PAGE and autoradiography. Chain truncation mutations are detected by the presence of a lower-molecular-weight (increased mobility) species relative to the wild-type (WT) protein band. More recent variants of the SDS-PAGE-based PTT include non-radioactive versions based on Western blotting [13] or, as reported by us, using tRNA-mediated protein engineering to fluorescently label the expressed protein [14]. Additional benefits of the PTT include (a) the potential to be one of the highest throughput mutation scanning methods since 1 to 3 kb of DNA sequence can be scanned from a single PCR, compared with 200 to 300 base pairs in many other methods [15,16], and (b) the ability to detect large genomic deletions and insertions as well as mRNA splicing errors that can be missed by methods such as direct DNA sequencing and DHPLC [16,17].

However, as with all of the electrophoresis-based methods, PTT is inherently low-throughput and difficult to automate, and results (which are based on visual inspection of the gel) can be difficult to interpret, thereby making them subject to human error [2]. Furthermore, mutations in the 5' end of the *BRCA *gene which result in small truncation fragments are missed (not resolved on gel) [16] and this affects the diagnostic sensitivity.

To overcome these limitations, we previously developed [14] the first high-throughput solid-phase protein truncation test (HTS-PTT), which uses an industry-standard microtiter-plate enzyme-linked immunosorbent assay (ELISA) format, which can be implemented in any clinical laboratory with minimal, inexpensive, and widely used instrumentation. This assay, which we previously applied to the detection of familial adenomatous polyposis [14], used a triple-tag system comprised of N-terminal and C-terminal epitope tags for normalization and mutation detection, respectively, as well as directly incorporated biotin labels as the capture tag for protein immobilization on the ELISA plate. Unfortunately, biotin incorporation during translation using tRNA-mediated protein engineering occurs at very low efficiency, thereby adversely affecting mutation detection capabilities and diagnostic sensitivity (data not shown). Du and colleagues [18] later introduced a two-tag variant of our assay as applied to the genetic disorder ataxia-telangiectasia; however, this variant lacks the ability to normalize for the amount of total nascent protein that is expressed and captured on the ELISA plate, critical for the reproducibility and accuracy required in a clinical or diagnostic setting or both.

**Figure 1:**
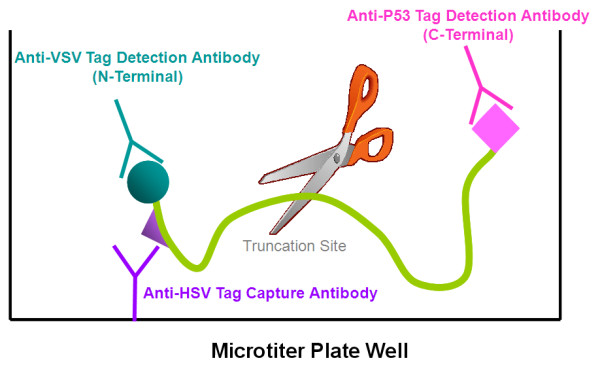
**Schematic representation of the high-throughput solid-phase protein truncation test (HTS-PTT)**. The next-generation HTS-PTT uses three different epitope tags - two in tandem at the N-terminus (one capture and one detection tag) and one at the C-terminus (detection tag) - added using specially designed primers (not shown here). The chain truncation mutations are calculated using % C/N (the C- to N-terminal signal ratio of a given sample normalized against known wild-type controls).

The next-generation HTS-PTT, reported here, uses three different epitope tags - two in tandem at the N-terminus (one detection and one capture tag) and one at the C-terminus (detection tag) - added by using specially designed PCR primers that amplify target gene segments from patient blood. The amplified DNA is cell-free expressed and simultaneously captured and purified by epitope capture onto the ELISA plate, and the remaining two tags enable N- and C-terminal detection (Figure 1). We have applied this improved assay for the first time to the detection of truncation mutations in exons 11 of the breast cancer susceptibility genes *BRCA1/2 *and have evaluated it using 100 clinical genomic DNA test samples.

## Materials and methods

Human samples were obtained through the Women's College Research Institute (ref. no. 2007-0036-B) for these experiments and for the collaboration with AmberGen, Inc. (Watertown, MA, USA). All study subjects provided informed written consent. For HTS-PTT analysis, purified genomic DNA samples were provided to AmberGen, Inc., in de-identified form, marked only with non-descriptive alphanumeric codes along with only the *BRCA1/2 *mutation status/designation, and no identifying information or clinical annotation was provided to AmberGen, Inc.

### DNA and polymerase chain reaction

One hundred human genomic clinical DNA samples isolated from blood and previously characterized by the conventional gel-based PTT (method as described in [19]) were used for HTS-PTT. The genomic DNA was extracted from peripheral blood leukocytes by means of the Gentra Puregene Blood Kit (Gentra Systems, Minneapolis, MN, USA). mRNA samples for HTS-PTT were extracted from several *BRCA *mutant cell lines (B-lymphocytes from blood) obtained from the Coriell Cell Repository (Camden, NJ, USA); samples were extracted by means of the Qiagen Rneasy Mini Kit (Qiagen, Germantown, MD, USA) and converted to cDNA by means of the SMART™ PCR cDNA Synthesis Kit (Clontech, Mountain View, CA, USA). PCR amplification was carried out with 50 ng of genomic DNA or cDNA, 0.245 μM each of the forward and reverse primers, 2.5 mM MgCl_2_, 0.2 mM dNTPs (each), and the Phusion Hot Start DNA Polymerase with the HF Buffer (New England Biolabs, Ipswitch, MA, USA). Amplification was performed as follows: an initial denaturation at 95°C for 2.5 minutes, 40 cycles of denaturation at 95°C for 30 seconds, annealing at 67.5°C for 45 seconds, extension at 72°C for 3.5 minutes, and a final extension step at 72°C for 10 minutes. The gene-specific PCR primers used on genomic DNA or cDNA templates were as follows:

Forward primer: 5'-ggATCC*TAATACgACTCACTATA*gggAgACCACCATg**TACACCgACATCgAgATgAACCgCCTgggCAAg**ggAggA**CAgCCTgAACTCgCTCCAgAggATCCggAAgAT**[gene-specific hybridization region]-3'.

Forward primer key: The italicized nucleotides correspond to the T7 promoter, the underlined ATg is the initiation codon, the boldface nucleotide region codes for the N-terminal detection tag (VSV-G; YTDIEMNRLGK), the underlined boldface nucleotide region codes for the N-terminal capture tag (HSV; QPELAPEDPED), the bracketed nucleotide sequence codes for the *BRCA*-specific complementary region (see below), and the remaining nucleotide sequences correspond to the Kozak (ribosome binding) and spacer regions.

Reverse primer: 5'-TTATTA**CAgCAgCTTgTgCAggTCgCTgAAggT **[gene-specific hybridization region]-3'.

Reverse primer key: The boldface nucleotides code for the C-terminal detection tag (p53-derived tag; TFSDLHKLL), the underlined TTATTA is two successive stop codons, and the bracketed nucleotide sequence codes for the *BRCA*-specific complementary region (see below).

**Figure 2:**
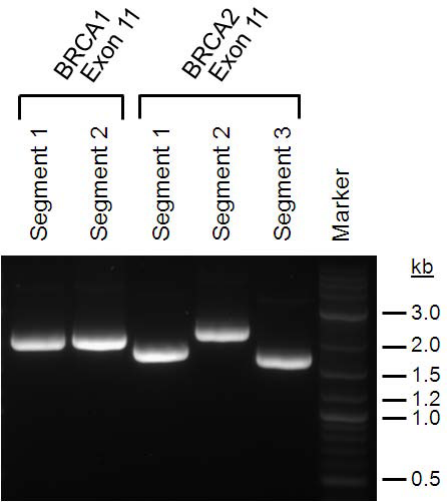
**Example of agarose gel analysis of five different polymerase chain reaction (PCR) segments spanning exons 11 of *BRCA1 *and *BRCA2***. The segment sizes correspond to the values in Table 1, demonstrating the quality and quantity of the PCR fragments. *BRCA1/BRCA2*, breast and ovarian cancer susceptibility gene 1/2.

**Figure 3:**
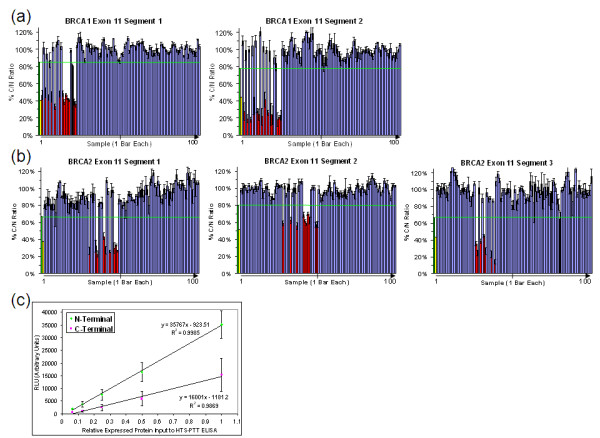
**Clinical validation of high-throughput solid-phase protein truncation test (HTS-PTT) on *BRCA1/2 *exons 11 for inherited breast cancer susceptibility**. One hundred blood-derived clinical genomic DNA samples were analyzed by HTS-PTT. **(a) ***BRCA1 *covered in two overlapping segments. **(b) ***BRCA2 *covered in three overlapping segments. **(c) **Standard curve of different protein inputs into the HTS-PTT enzyme-linked immunosorbent assay (ELISA) using serial dilutions of known wild-type samples. % C/N is the C- to N-terminal signal ratio of a given sample normalized against known wild-type controls. The green bars and lines indicate the designated threshold (3 standard deviations below the mean for the wild-type cohort), above which samples are scored as wild-type (blue bars) and below which samples are scored as mutant (red bars). Yellow bars indicate the predicted mutant % C/N based on the known wild-type standard curves (c). Black bars denote false-positives. The tick mark on the x-axis divides the 50 patients with known *BRCA1/2 *truncation mutations (left of tick mark) from the 50 wild-type patients (right of tick mark). *BRCA1/BRCA2*, breast and ovarian cancer susceptibility gene 1/2; RLU, relative light unit.

Gene-specific hybridization regions and sequence of the primers are provided in Table 1. After amplification, the quality and quantity of the PCR products were analyzed by agarose gel electrophoresis. Refer to Figure 2 for an example of agarose gel analysis of the five different PCR segments, and refer to Figure 3 for the names of the aforementioned cell lines used for the mRNA samples.

**Table 1:** *BRCA1/2 *exon 11 primers and segments for high-throughput solid-phase protein truncation test

Segment	Nucleotides	Segment size, base pairs	Primer sequence (5' to 3') F, forward; R, reverse; subscript 'I', intronic primer
*BRCA1*^a^			
1	123,126-125,033	1,908	F: gCTTgTgAATTTTCTgAgACggAT
			R: TgTATTCTgCAAATACTgAgCATCAAg
2	124,617-126,578	1,962	F: gTCAATCCTAgCCTTCCAAgAgAA
			R_I_: gggCAAACACAAAAACCTggTTCC
*BRCA2*^a^			
1	25,758-27,458	1,701	F_I_: TTTTgTCACTTTgTgTTTTTATgTTTAgg
			R: gCCAgCAAACTTCCgTTTAATTTC
2	27,174-29,330	2,157	F: AACCATAATTTAACACCTAgCCAAAAg
			R: TgAAgAgCTAgTCACAAgTTCCTC
3	29,181-30,743	1,563	F: gATTCTggTATTgAgCCAgTATTgAAg
			R_I_: CACgAAAggTAAAAATgAACACTTACC

^a^National Center for Biotechnology Information (NCBI) reference sequences: Ng_005905.2 for *BRCA1 *and Ng_012772.1 for *BRCA2*, both numbered from whole sequence entry. *BRCA1/BRCA2*, breast and ovarian cancer susceptibility gene 1/2.

### Cell-free protein synthesis

The rabbit reticulocyte cell-free reaction mixture contained 8.5 μL of TNT T7 Quick Rabbit Reticulocyte Lysate for PCR DNA (Promega, Madison, WI, USA), 0.5 μL of a complete amino acid mix (50 μM final for each), and 1 μL of PCR-amplified DNA (approximately 100 to 200 ng). The coupled transcription-translation reaction was allowed to proceed for 45 minutes at 30°C.

### HTS-PTT

MicroLite 2 + 96-well microtiter plates (Thermo Fisher Scientific, Waltham, MA, USA) were pre-coated with 0.5 ng/μL of an anti-HSV tag monoclonal antibody (EMD Biosciences, La Jolla, CA, USA) solution in 50 mM sodium carbonate at a pH of 9.5. The plates were then washed four times with Tris-buffered saline (TBS) containing 0.05% (vol/vol) Tween-20 (TBS-T) and blocked for 30 minutes with TBS-T containing 1% (wt/vol) bovine serum albumin (BSA). After protein synthesis, the reaction mixture was diluted 100-fold with TBS-T further supplemented with 0.1% Triton X-100, 1% (wt/vol) BSA, and one of either the N- or C-terminal detection antibodies (anti-VSV-G-HRP at 40 ng/mL, Clone P5D4, Roche Applied Science, Indianapolis, IN, USA; or anti-p53-HRP at 80 ng/mL, Clone BP53-12, Santa Cruz Biotechnology, Inc., Santa Cruz, CA, USA; HRP = horseradish peroxidase). Alternatively, both the anti-VSV-G-HRP antibody and an anti-p53-AP antibody (Santa Cruz Biotechnology, Inc.; AP = alkaline phosphatase) could be used in the same solution (same concentrations as above) to ultimately allow both signals to be detected in the same well using this dual-reporter system [14]. In either case, 100 μL of the diluted reaction mixture was then added to each well of the aforementioned antibody-coated microtiter plates and incubated for 45 minutes on an orbital shaker. The plate was washed four times with TBS-T and developed using a chemiluminescent HRP substrate (Super Signal Femto; Pierce Chemicals, Rockford, IL, USA). If the dual-reporter system was used, a chemiluminescent AP substrate was added first (Roche Applied Science), and after signal readout, the plates were washed four times with TBS-T and developed using the HRP substrate described above. All signals were read using a LumiCount luminescent plate reader (Packard Biosciences, Meriden, CT, USA). The background levels correspond to cell-free protein synthesis reactions that lacked only the added DNA in the reaction mixture.

## Results and Discussion

### The basic HTS-PTT

A schematic of the HTS-PTT assay is shown in Figure 1 (PCR and expression not shown). *BRCA1/2 *open reading frames are amplified and divided into 'working' segments using PCR (corresponding to roughly 50- to 75-kDa overlapping protein segments within exons 11 of *BRCA1 *and *BRCA2*; Table 1). As detailed in the Materials and methods section, the PCR also incorporates all of the needed expression sequences (for example, promoter) and epitope tag sequences, as required by the HTS-PTT method. After cell-free coupled transcription/translation of the PCR products, the nascent proteins are analyzed by HTS-PTT for truncation mutations. The N-terminal capture tag (HSV epitope) is used for concurrent immobilization/purification of the cell-free expressed protein onto antibody-coated microtiter-well ELISA plates. The N- and C-terminal detection tags (VSV-G and p53 epitopes, respectively) are subsequently used for measuring the relative level of shortened protein produced by the chain truncation mutation (Figure 1). Detection is achieved using HRP-labeled epitope tag antibodies and a highly sensitive chemiluminescent readout in separate replicate wells of the ELISA plate. Alternatively, if tighter control is desired, a per-well normalization is possible, in which N- and C-terminal epitope tag antibodies (each carrying a different enyzyme reporter) are used in the same wells [14].

### Polymerase chain reaction optimizations on *BRCA1/2 *for HTS-PTT

Although lengthy sequences need to be added by the PCR primers in the HTS-PTT process, we have fully optimized the PCR conditions so that this can be achieved in a single reaction (one-step PCR) with a single primer pair (approximately 130-mer forward and approximately 60-mer reverse) as opposed two sequential PCRs commonly used to add sequences of this length to an amplicon. This is important since two sequential PCRs are not compatible with standard clinical PCR clean-room practices (that is, separate pre- and post-PCR rooms). A significant problem we encountered is that the lengthy one-step PCR primers are prone to form non-specific or primer dimer extension products (or both) that produce substantial background in the HTS-PTT since both can be expressed as protein and can contain the in-frame epitope tags. Optimizing factors such as primer concentration, annealing temperature, magnesium concentration, and the use of a heat-activated 'hot start' DNA polymerase were found to be critical in avoiding this problem (data not shown). Extreme care should be taken to avoid the primer dimer extension products in the one-step and even the two-step PCR methodologies as these products are ideal expression templates and can interfere with HTS-PTT even when present at levels near or below the detection limit of conventional ethidium bromide agarose gel electrophoresis. For instance, the quantity and quality of the genomic DNA input into the PCR should be closely monitored (discussed later) as, in general, less cognate template means that more primer dimer extension products will be formed. All experiments described in this article use this optimized one-step PCR method (see Figure 2 for an example of agarose gel analysis of five different PCR segments).

### Linearity of the HTS-PTT assay

After the HTS-PTT assay (Figure 1), the C-terminal signal arising from full-length protein in a given sample is normalized against its N-terminal signal representing total nascent protein produced and is captured on the ELISA plate. These C-terminal-to-N-terminal signal ratios (C/N ratios) of the test samples are calculated as a percentage of the ratio arising from known *BRCA *WT control samples (% C/N). Thus, in theory, *BRCA *WT samples would have a 100% C/N ratio and heterozygous *BRCA *mutants would have a 50% ratio.

In practice, however, the % C/N ratios of the *BRCA *heterozygous mutants deviate from 50% in either direction (for example, Figure 3a and 3b; red bars for various segments), a phenomenon that can be explained by several possible factors. All of these factors essentially derive from the coexistence of two protein species in the heterozygous mutant samples (full-length and truncated) as opposed to a single protein species in the WT samples (full-length). One likely factor is skewed ratios of the actual mutant and WT proteins within a heterozygous sample, either in the expressed sample itself or ultimately what is captured on the ELISA plate, and this could occur through various mechanisms. For example, the enhanced expression or plate-binding kinetics of the mutant protein, owing to its decreased size compared with the WT [14], could increase the ratio of mutant protein actually bound to the ELISA plate to greater than 50% and therefore skew the % C/N ratio to less than 50%. Similarly, nucleotide composition differences between the mutant and WT can ultimately alter the mRNA secondary structure and hence result in differences in expression efficiency between the mutant and WT, and this may be particularly applicable to larger deletions or insertions and could skew the % C/N in either direction. Differences in the physical accessibility (that is, steric hindrance) of the N-terminal epitope tag between the mutant and WT proteins could be caused by different secondary and tertiary folding structures between the two protein species and thereby could skew the ratio of mutant and WT protein captured on the ELISA plate.

Importantly, these effects could vary among the different *BRCA *test segments as well as from sample to sample (depending on the position of the mutation). As seen in Figure 3a and 3b (red bars; mutant samples), the former is the overriding factor as each *BRCA *segment tends to have a unique % C/N ratio for the mutants. For example, *BRCA2 *exon 11 segment 1 mutants fall in the range of 30% ± 6% C/N, whereas *BRCA2 *exon 11 segment 2 mutants fall in the range of 61% ± 5% C/N (Figure 3b). However, intra-segment variation in the % C/N ratio for the mutants is also apparent.

Figure 3c shows an example of linearity of the HTS-PTT assay as a function of different amounts of translated protein input into the ELISA assay. The standard curves in Figure 3c are for the N- and C-terminal detection tags of the WT reference sample for *BRCA2 *exon 11 segment 1 (*n *= 7). Importantly, although this verified excellent linearity of the assay over an approximate 10-fold range (R^2 ^= 0.99), the y-intercepts of these N- and C-terminal signal curves do not pass precisely though zero. A critical consequence of this is that, in order to correctly compare the % C/N ratio of the WT reference sample with that of the test samples, the input amount of translated protein (for the ELISA) must be standardized among all of the samples. This is easily and typically achieved by normalizing the input amount of PCR product into the cell-free protein expression reaction and can be quality-controlled by monitoring the raw N-terminal signals (total expressed and captured protein).

### Clinical validation of a genomic DNA-based HTS-PTT on *BRCA1/2 *using a 100-member training sample set

To evaluate the HTS-PTT for *BRCA *mutation analysis on clinical samples, we designed primer pairs to fully cover the large exons 11 of both *BRCA1 *and *BRCA2*, dividing them into two and three overlapping segments, respectively; each segment was roughly 2 kb in size. One hundred clinical genomic DNA samples collected from blood were analyzed by means of HTS-PTT (25 *BRCA1 *mutants, 25 *BRCA2 *mutants, and 50 normal controls). The mutation status of the samples had been previously determined by conventional gel-based PTT. HTS-PTT results are shown in Figure 3a and 3b. The range of mutations covered is detailed in Supplemental table S1 in Additional file 1. This sample cohort contained deletions as large as 40 bases, all of which were detected by our HTS-PTT method.

Despite the aforementioned segment-specific % C/N ratios for heterozygous mutants in HTS-PTT, a fixed method based on the variability within the WT cohort was still able to be employed for setting the diagnostic scoring cutoffs. HTS-PTT scoring cutoffs for a positive protein truncation were fixed at 3 standard deviations below the mean of the 50 normal (WT) samples (on a segment-by-segment basis) for an approximately 99% confidence interval. With these cutoffs (Figure 3a and 3b; green bar and green line), the sensitivity and specificity of the HTS-PTT (as compared with conventional PTT) were 100% and 96%, respectively. Note that the % C/N ratios for the only two false-positive calls (Figure 3a and 3b; black bars) were just slightly below the cutoff and were likely due to assay variance (approximately 1/100 false-positive calls are statistically expected with the aforementioned 99% confidence interval used). Finally, it is worth noting that, in the HTS-PTT assay, raw signal-to-noise ratios of the N- and C-terminal detection were never less than 20:1 and in most cases were greater than 100:1.

### An mRNA-based HTS-PTT for *BRCA1/2*

**Figure 4:**
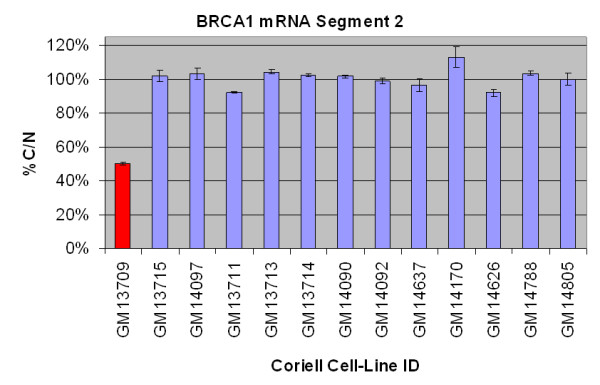
**Example of *BRCA1 *mRNA-based high-throughput solid-phase protein truncation test (HTS-PTT)**. mRNA extracted from 13 cell-line samples was analyzed by means of the HTS-PTT. % C/N is the C- to N-terminal signal ratio of a given sample normalized against known wild-type controls. The mutant sample is designated by the red bar. *BRCA1/BRCA2*, breast and ovarian cancer susceptibility gene 1/2.

To obtain full *BRCA1/2 *(open reading frame) coverage using a genomic DNA test, roughly 50 HTS-PTT segments per patient would be required because of the large number of small exons in both genes, outside of the large exons 11. The high-throughput ELISA-based HTS-PTT is certainly more amenable to such large segment numbers than the conventional gel-based PTT. However, one way to further alleviate this issue is to design an mRNA-based assay, which would allow the *BRCA1/2 *transcripts to be divided evenly into approximately 10 HTS-PTT segments of roughly 1 to 2 kb in size (an effective size for the HTS-PTT). To demonstrate the feasibility of this approach, we performed experiments on cDNA created from cell lines (obtained from the Coriell Cell Repository) having known *BRCA1/2 *truncating mutations (Coriell Institute for Medical Research, Camden, NJ, USA). Figure 4 shows *BRCA1 *results for one gene segment on 13 cell lines (1 mutant) and again demonstrates 100% accuracy. Importantly, nonsense-mediated decay of the mutant transcripts was not found to be a problem in this small set, but should the problem occur, mutation detection sensitivity as low as 25% is possible with HTS-PTT [14].

## Conclusions

The HTS-PTT has the potential to cover the vast majority of clinically important *BRCA1/2 *mutations (approximately 90% of BIC entries) using a rapid and facile microtiter-plate ELISA assay. The assay avoids radioactivity as well as very low-throughput SDS-PAGE analyses and, unlike SDS-PAGE-based PTT, uses concrete mathematical thresholds to distinguish mutant and WT samples. Here, in *BRCA1/2 *exons 11, clear thresholds could be established to provide 100% diagnostic sensitivity and 96% specificity for truncating mutations in these 100 clinical genomic DNA samples. Furthermore, an mRNA-based HTS-PTT shows the feasibility of covering all of *BRCA1/2 *and would require about 10 segments. Overall, the assay affords a simple, low-cost, and high-throughput method of *BRCA *analysis that would be readily accessible to virtually any laboratory with rudimentary instrumentation (PCR machine and basic ELISA plate reader).

## Abbreviations

BIC: Breast Cancer Information Core; *BRCA1/BRCA2*: breast and ovarian cancer susceptibility gene 1/2; BSA: bovine serum albumin; C/N: C-terminal to N-terminal signal (ratio); DHPLC: denaturing high-performance liquid chromatography; ELISA: enzyme-linked immunosorbent assay; HRP: horseradish peroxidase; HTS-PTT: high-throughput solid-phase protein truncation test; PCR: polymerase chain reaction; PTT: protein truncation test; TBS: Tris-buffered saline; TBS-T: Tris-buffered saline containing 0.05% (vol/vol) Tween-20; WT: wild-type.

## Competing interests

MJL, HPO, and GJF are current employees of AmberGen, Inc., a developer of commercial diagnostic assays. SG is a former employee (within the last 5 years), and KJR is a co-founder of the company. This project was financed by AmberGen, in part using Small Business Innovation Research grant funds (from the National Institutes of Health) awarded to AmberGen (see Acknowledgements). AmberGen is the assignee on issued patents related to the commercial use of the HTS-PTT technologies described in this article. However, these patents do not preclude the use of the HTS-PTT by the general research community. In the article, we disclose in full detail the methodology required for researchers to perform HTS-PTT, which needs no specialized reagents or instrumentation (and requires nothing to be purchased from AmberGen).

## Authors' contributions

MJL and KJR helped conceive of the HTS-PTT assay and its application to *BRCA *analysis, participated in the design and coordination of all studies in this article, and contributed significantly to the drafting of the manuscript. SG helped conceive of the HTS-PTT assay and its application to *BRCA *analysis and participated in the design and coordination of all studies in this article. SN supervised and coordinated the isolation and gel-PTT analysis of all clinical genomic DNA samples used in this article and contributed significantly to the drafting of the manuscript. GJF was responsible for performing and designing PCR amplifications, cell-free protein expressions, and HTS-PTT assays for optimization purposes and for the final analyses of the clinical samples. HPO performed advanced PCR assays that contributed to a better understanding of the effects of primer dimers and non-specific PCR extension products on the HTS-PTT assay for *BRCA *analysis and contributed significantly to the drafting of the manuscript. All authors read and approved the final manuscript.

## Supplementary Material

Additional file 1 **Supplemental table S1 - *BRCA1/2 *Mutations Covered by the HTS-PTT**. This table lists the mutation designations for the 50 patient genomic DNA samples tested which were positive for *BRCA1/2 *truncation mutations. HTS-PTT segments containing the mutation and the measured % C/N ratios are also listed. Click here for file
