# Supplementary material for: An ELISA-based high throughput protein truncation test for inherited breast cancer
Source: Breast Cancer Res. 2010 Oct 4;12(5):R78. doi: 10.1186/bcr2722 (PMC3096971; doi:10.1186/bcr2722)
Supplement: Additional file 1 — Supplemental table S1 - BRCA1/2 Mutations Covered by the HTS-PTT. This table lists the mutation designations for the 50 patient genomic DNA samples tested which were positive for BRCA1/2 truncation mutations. HTS-PTT segments containing the mutation and the measured % C/N ratios are also listed. [file bcr2722-S1.DOC]

**Supplemental Table 1 – BRCA1/2 Mutations Covered by the HTS-PTT**

| **Patient ID (BRCA1 Mutations)** | **Mutation*** | **HTS-PTT Segment** | **% C/N Ratio** |
| --- | --- | --- | --- |
| 1 | BRCA1 exon11 1479delAG | 1 | 47 |
| 2 | BRCA1 exon11 3875delGTCT | 2 | 28 |
| 3 | BRCA1 exon11 2072del4 | 1 | 49 |
| 4 | BRCA1 exon11 3118delA | 2 | 19 |
| 5 | BRCA1 exon11 A1506G,1510insG | 1 | 44 |
| 6 | BRCA1 exon11 3118delA | 2 | 18 |
| 7 | BRCA1 exon11 1479delAG | 1 | 45 |
| 8 | BRCA1 exon11 2953delGTAinsC | 2 | 34 |
| 9 | BRCA1 exon11 1293del40 | 1 | 33 |
| 10 | BRCA1 exon11 2953del2 | 2 | 29 |
| 11 | BRCA1 exon11 3092del7 | 2 | 24 |
| 12 | BRCA1 exon11 1014delGT | 1 | 48 |
| 13 | BRCA1 exon11 2681insGC | 2 | 22 |
| 14 | BRCA1 exon11 1048delA | 1 | 48 |
| 15 | BRCA1 exon11 2524delTG | 1 and 2 | 38 and 42 |
| 16 | BRCA1 exon11 1479delAG | 1 | 47 |
| 17 | BRCA1 exon11 C2457T | 1 and 2 | 42 and 26 |
| 18 | BRCA1 exon11 1294del40 | 1 | 41 |
| 19 | BRCA1 exon11 C3726T | 2 | 24 |
| 20 | BRCA1 exon11 2953delGTAinsC | 1 and 2 | 81^¶^ and 23 |
| 21 | BRCA1 exon11 A2154T | 1 | 38 |
| 22 | BRCA1 exon11 2190delA | 1 | 36 |
| 23 | BRCA1 exon11 3450del4 | 2 | 18 |
| 24 | BRCA1 exon11 C3726T | 2 | 21 |
| 25 | BRCA1 exon11 3407delAA | 2 | 23 |
|  |  |  |  |
| **Patient ID (BRCA2 Mutations)** | **Mutation*** | **Segment** | **% C/N Ratio** |
| 26 | BRCA2 exon11 6174delT | 3 | 36 |
| 27 | BRCA2 exon11 C5910G | 3 | 24 |
| 28 | BRCA2 exon11 5302insA | 2 | 59 |
| 29 | BRCA2 exon11 6174delT | 3 | 39 |
| 30 | BRCA2 exon11 3036delACAA | 1 | 27 |
| 31 | BRCA2 exon11 5700insA | 3 | 45 |
| 32 | BRCA2 exon11 6503delTT | 3 | 27 |
| 33 | BRCA2 exon11 4706del4 | 2 | 63 |
| 34 | BRCA2 exon11 2816insA | 1 | 32 |
| 35 | BRCA2 exon11 3034delAAAC | 1 | 23 |
| 36 | BRCA2 exon11 6633del5 | 3 | 23 |
| 37 | BRCA2 exon11 4206ins4 | 2 | 56 |
| 38 | BRCA2 exon11 G6085T | 3 | 14 |
| 39 | BRCA2 exon11 2515delC | 1 | 44 |
| 40 | BRCA2 exon11 3034delAAAC | 1 | 27 |
| 41 | BRCA2 exon 11 4866 delT | 2 | 69 |
| 42 | BRCA2 exon11 5573del AA | 2 | 62 |
| 43 | BRCA2 exon11 3772delTT | 1 and 2 | 26 and 58 |
| 44 | BRCA2 exon11 A5002T | 2 | 70 |
| 45 | BRCA2 exon11 4075delGT | 2 | 62 |
| 46 | BRCA2 exon11 3398del5 | 1 | 30 |
| 47 | BRCA2 exon 11 T2637G | 1 | 34 |
| 48 | BRCA2 3522delT | 1 | 26 |
| 49 | BRCA2 exon11 4075delGT | 2 | 57 |
| 50 | BRCA2 exon11 4075delGT | 2 | 57 |

* Mutation numbering is based on the reference mRNA sequences provided in the BIC (GenBank: U14680.1 for BRCA1 and GenBank: U43746.1 for BRCA2)

¶ 81% C/N ratio represents a false-positive for this segment, although Patient 20 does have a chain-truncating mutation in Segment 2.
